# Thermal discomfort with cold extremities in relation to age, gender, and body mass index in a random sample of a Swiss urban population

**DOI:** 10.1186/1478-7954-8-17

**Published:** 2010-06-04

**Authors:** Maneli Mozaffarieh, Paola Fontana Gasio, Andreas Schötzau, Selim Orgül, Josef Flammer, Kurt Kräuchi

**Affiliations:** 1 University Eye Clinic, Basel, Switzerland; 2 Thermophysiological Chronobiology, Centre for Chronobiology, Psychiatric University Clinics, Basel, Switzerland

## Abstract

**Background:**

The aim of this epidemiological study was to investigate the relationship of thermal discomfort with cold extremities (TDCE) to age, gender, and body mass index (BMI) in a Swiss urban population.

**Methods:**

In a random population sample of Basel city, 2,800 subjects aged 20-40 years were asked to complete a questionnaire evaluating the extent of cold extremities. Values of cold extremities were based on questionnaire-derived scores. The correlation of age, gender, and BMI to TDCE was analyzed using multiple regression analysis.

**Results:**

A total of 1,001 women (72.3% response rate) and 809 men (60% response rate) returned a completed questionnaire. Statistical analyses revealed the following findings: Younger subjects suffered more intensely from cold extremities than the elderly, and women suffered more than men (particularly younger women). Slimmer subjects suffered significantly more often from cold extremities than subjects with higher BMIs.

**Conclusions:**

Thermal discomfort with cold extremities (a relevant symptom of primary vascular dysregulation) occurs at highest intensity in younger, slimmer women and at lowest intensity in elderly, stouter men.

## Introduction

There are many individuals worldwide who often have cold extremities. Cold extremities are a leading symptom for a variety of syndromes. In young individuals, cold extremities are normally a symptom of the complex syndrome known as primary vascular dysregulation (PVD) [1]. The various terminologies for PVD, all of which describe more or less the same syndrome, include "vasospastic syndrome" [2], "neurasthenia"[3], "neuro-vegetative dystonia"[4], and "hi-e sho" in Japan [5].

Subjects with PVD have vascular systems that respond differently, explaining the terminology PVD. However, they also exhibit other differences, such as in their sleep behavior [6], feeling of thirst [7], or sensitivity to medication [8]. PVD occurs more frequently in females than in males, in Japanese than in Caucasians, and in academics than in blue-collar workers [9]. The symptoms are normally first manifested in puberty and mitigate with age. In females, a marked reduction in symptoms is often observed after menopause but can re-exacerbate when treated with hormonal replacement therapy [10].

Cold extremities are normally a sign of reduced blood flow [11]. Blood flow can be reduced due to diseased vessels (e.g., arteriosclerosis) or due to dysregulation. The former occurs more often in the elderly, whereas the latter occurs more often in the young, particularly in young females. One aspect of a dysregulation is called vasospasms [9]. Vasospasms are defined as inappropriate vasoconstrictions often confined to short segments of the vessels (e.g., at a bifurcation of an artery). Dysregulation, however, also implies insufficient dilation of vessels when needed, as in the context of neurovascular coupling. In addition, it can imply an inappropriate dilation, particularly in the venous side. A vascular dysregulation can occur in any organ. Dysregulation is indeed well documented in the heart [12] and particularly occurs in the extremities. In the eye, PVD can lead to disturbed autoregulation of ocular blood flow [13].

The purpose of this epidemiological study was to investigate the relationship between thermal discomfort with cold extremities (TDCE) and age, gender, and body mass index (BMI) in a random sample of a Swiss urban population. The present multivariate analysis covers these further aspects of a previously published study of the same survey [14].

## Methods, data management

A random sample of 2,800 men and women aged 20-40 years was selected from the population register of Basel city, Switzerland. The study was approved by the ethical committee of Basel city and Basel country. A postal questionnaire about the intensity of cold extremities was sent to all subjects [14]. In order to calculate a score, the following two questions were asked: (A) During the past month, how intensely did you suffer from cold hands? (B) During the past month, how intensely did you suffer from cold feet? The possible answers were: 1 = 'not at all', 2 = 'a little', 3 = 'quite', 4 = 'extraordinary'. A sum of the two questions was calculated (range: 2-8). In a previous study, TDCE was externally validated using objective skin temperature measurements (TASCO infrared thermometer THI-500, Osaka, Japan) [15]. In order to visualize the findings, BMI was categorized into six percentiles (0-10% = 15.5-19.0 kg/m^2^; 10-25% = 19.0-20.2; 25-50% = 20.2-22.1; 50-75% = 22.1-24.4; 75-90% = 24.4-27.2; 90-100% = 27.3-61.4). Age was categorized into seven three-year bins.

### Statistical Analysis

To predict TDCE from age, BMI, and gender, a linear regression model was performed. The dependent variable was TDCE, and the independent variables were age, BMI, and gender, as well as all two- and three-way interactions. To adjust for possible effects from smoking, disease, or contraceptive use, these variables were included in the regression model. Regression results are reported as differences of means for categorical variables or differences increasing the predictor one unit for metric variables.

A 95% confidence interval and p-values were also reported. (A p-value < 0.05 is considered significant.)

All analyses were done using R version 2.8.1 (A Language and Environment for Statistical Computing).

## Results

A total of 1,001 women (72.3% response rate reached in two waves) and 809 men (60% response rate reached in three waves) returned a completed questionnaire. No significant differences in the results were found among the three waves. Women were over-represented in the study population (55% women versus 45% men) compared to the population of Basel city (51% versus 49%); the results are presented separately for women and men. The distribution of age and BMI within the gender groups did not differ statistically from the distribution in the population of Basel city (comparisons carried out with data of the annual statistical report of the canton Basel city 2004; data not shown) indicating a representative sample in these respects.

**Figure 1:**
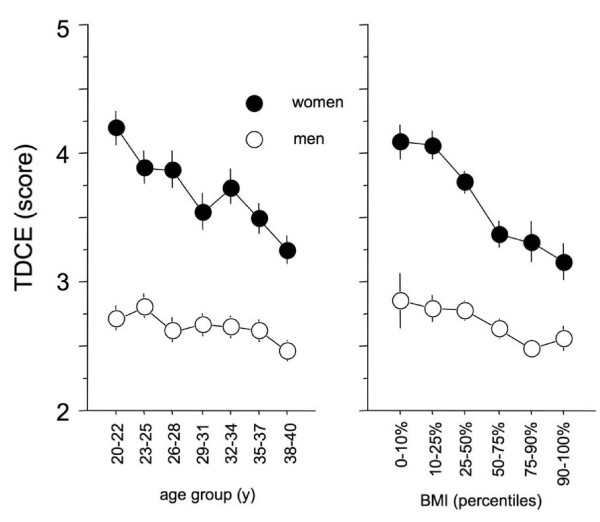
**The left panel depicts the relationship of cold extremities to age and gender, showing that women in general, and particularly young women, exhibit higher intensity of cold extremities than men**. The right panel depicts the relationship of BMI to gender showing that slimmer women exhibit higher intensity of cold extremities than men. Values are given in mean ± sem.

The regression analysis using TDCE as the dependent variable revealed a significant interaction between gender and BMI (p = 0.004), as well as gender and age (p = 0.03). This indicates different dependencies of TDCE with respect to BMI and age for each gender (Figure 1). Separate regression models for each gender were performed (Table 1). Women exhibit a stronger dependency of TDCE on age and BMI than men.

**Table 1:** Separate regression models for each gender

Female	Difference*	**95% C.I**.	p-value
AGE	-0.040	-0.056 - -0.025	<0.001
BMI	-0.059	-0.083 - -0.036	<0.001

**Male**	Difference*	**95% C.I.**	**p-value**
AGE	-0.011	-0.022 - 0.000	0.0533
BMI	-0.023	-0.042 - -0.004	0.0181

* Difference increasing the predictor variable by one unit (e.g., TDCE decreases by 0.04 units when age increases by 1 year)

This indicates younger subjects suffered more intensely from cold extremities than the elderly, and women suffered more than men (Figure1 left panel). Furthermore, slimmer subjects suffered significantly more often from TDCE in comparison to subjects with higher BMI (Figure1 right panel). All other interactions were not significant (p > 0.5).

Additionally, there is a strong gender difference (Table 2; main effect difference of means = 0.99, C.I.= 0.864 - 1.113, p < 0.001, adjusted to mean BMI and mean age).

**Table 2:** Means (sd) or counts (%) split by gender

	MALE	FEMALE
TDCE	2.649(0.962)	3.720(1.558)
AGE	30.1(6.0)	29.8(6.2)
BMI	23.84(3.60)	21.99(4.02)
SMOKE	304(37.5%)	357(35.4%)
DISEASE	251(31.0%)	397(39.3%)
CONTRACEPTIVES		342(34.2%)

SMOKE: number of subjects who smoke. DISEASE: number of subjects experiencing symptoms of illness in the last four weeks. CONTRACEPTIVES: number of women taking contraceptives.

Including smoking, disease, and contraceptive use in the regression model as additional covariates does not change results substantially (results not shown). Smokers and ill subjects exhibited higher TDCE (difference of means between smokers and nonsmokers = 0.26, C.I.= 0.13 - 0.39, p < 0.001; difference of means between ill and healthy subjects = 0.30, C.I. = 0.17 - 0.44, p < 0.001).

Table 2 shows the descriptive statistics of all relevant variables. The high proportion of ill persons in the study population was due to a high incidence of flu symptoms (approximately 50% of subjects with ill symptoms).

## Discussion

Various factors are believed to be associated with sensitivity to cold [16-18]. Of these, we focused on gender, age, and body mass index (BMI).

First, we found that women had significantly higher TDCE scores than men. Our results confirm the first rough data analysis showing that nearly every third woman between 20 and 40 years suffers from cold extremities -- with men suffering 4.5 times less frequently [14]. Numerous investigators have reported that women have colder mean skin temperatures during rest in cold air than men [19-22]. While this has been attributed to differences in body fat by some authors [20,21], others have not been able to show a relationship between fatness and mean skin temperature [23,24]. The literature is thus not in agreement.

Second, we found that young women particularly exhibited high TDCE scores. Our results are similar to those from a population-based study on people in Japan, where a standardized health questionnaire was used to obtain information on sensitivity to cold [25]. It was shown that a significantly higher percentage of women were sensitive to cold in comparison to men. Moreover, the percentage of women with sensitivity to cold did not increase with age.

Third, we found that individuals with a lower BMI suffered more from TDCE. This relationship was found even with the rather rough measure of body fatness (BMI). The degree of body fatness has been reported to influence one's ability to thermoregulate in a cold environment [26,27]. Body temperature is normally maintained by a complex series of mechanisms that control the production of body heat and the exchange of heat with the environment. Once the body's insulative capacity and peripheral vasoconstriction responses have been maximized, humans exposed to cold must rely on their ability to increase metabolism to counteract body heat loss and prevent hypothermia. These findings essentially did not change when the possible confounding factors of smoking, disease, and contraceptive use were included in the analysis, underlining the robustness of these relationships.

Variations in body proportions such as surface area to body mass or distributions of length proportions may also in part reflect how humans respond to climate. More than a century ago, Joel Asaph Allen made the interesting observation that animals living in cold environments have shorter limbs on average than animals living in warmer areas ("Allen's rule") [28]. This indicates that animals living in warmer environments need a larger surface for heat loss than those living in colder areas [29]. Allen's rule seems to be applicable to humans: When limb length between Yayoi and Jomon people of Japan were compared, Yayoi people, living in colder environments and higher altitudes, were shown to have significantly shorter relative limb lengths [29].

PVD subjects suffer more intensely from cold hands and feet (TDCE) [30,31]. These people also tend to have a low BMI. PVD occurs more often in women than men. The fact that the symptom manifests in puberty and decreases with age indicates that hormones, in particular estrogen, play a role. This explains why the syndrome can aggravate when estrogen is substituted after menopause [32]. However, the time courses of TDCE between 20 and 40 years are not in agreement with an exclusive hormonal explanation. In a very recent study, we could show that anger suppression in women associated with stereotypic feminine gender socialization seems to be an additional socio-psychological factor in the genesis of TDCE [33]. Men with PVD tend to more often suffer from serous chorioretinopathy. Testosterone most likely plays a role in this manifestation [34]. PVD subjects have a reduced feeling of thirst [7]. They tend to have low blood pressure, especially when they are young [35] (due to reduced reabsorption of sodium in the proximal tubule of the kidneys). They have a longer sleep onset latency [36,37], they more often suffer from migraines than non-PVD subjects [38], and often have an altered drug sensitivity due to differential expression of ABC transporter proteins [8]. They also exhibit a lower heart rate variability with a higher sympathovagal balance [39]. PVD subjects have a higher sensitivity for certain groups of drugs such as calcium channel blockers and systemic beta-blockers. This means that they require lower doses of these drugs to achieve the same effects and to avoid side effects. In contrast, their sensitivity is normal or rather decreased for other drugs, such as pain-killers.

In conclusion, we found that thermal discomfort with cold extremities occurs at highest intensity in younger, slimmer women and lowest in elderly, stouter men. Although this study reveals a clear statistical relationship between suffering from cold extremities to young age, female gender, and low BMI, it does not conclude whether there is a causal relationship.

## Competing interests

The authors declare that they have no competing interests.

## Authors' contributions

MM drafted the manuscript and assisted in statistical analysis.

PFG carried out the study and performed data processing.

AS performed the statistical analysis.

SO and JF provided advice on the study design and assisted in drafting the manuscript.

KK conceived the study, assisted in drafting the manuscript and performed data processing.

All authors read and approved the final manuscript.
